# Porous Thermoelectric Materials

**DOI:** 10.3390/ma2030903

**Published:** 2009-08-05

**Authors:** Hiroshi Julian Goldsmid

**Affiliations:** School of Physics, University of New South Wales, Sydney, NSW 2052, Australia; E-Mail: hjgoldsmid@phys.unsw.edu.au; Tel. +61-3-6229-1776

**Keywords:** thermoelectricity, porosity, bismuth telluride

## Abstract

Thermoelectric materials are sometimes prepared using a sintering process in which the achievement of a high density is often one of the objectives. However, it has recently been shown that the introduction of a highly porous material is desirable in synthetic transverse thermoelements. Porosity may also be an advantage in conventional longitudinal thermoelectric modules in which a high thermal flux density creates problems, but heat transfer within the pores can degrade the thermoelectric figure of merit. The amount of this degradation is calculated and it is shown that it can be small enough to be acceptable in practical devices.

## 1. Introduction 

The theory of thermoelectric energy conversion shows that the performance depends on the ratio of electrical to thermal conductance in each of the thermoelements [1]. It is usually assumed that the thermoelectric material is fully dense, as indeed it invariably is when it is grown from the melt. However, it is common practice to produce the material by a sintering process [2] and the possibility then exists of a degree of porosity. 

At first sight it might appear that porosity should not affect the performance since it should not change the ratio of electrical to thermal conductance, if there is no transport of electricity or heat within the pores. In fact, there may well be an advantage in reducing both conductances by the same proportion. The cost of material is a significant factor for the manufacturers of thermoelectric modules and there is a trend towards the use of thermoelements of reduced length and cross-section [3]. However, the consequent increase in the electric current density makes any contact resistance at the junctions of greater significance. Furthermore, the higher thermal flux density enlarges the unwanted temperature differences between the module and the source and sink. Both these problems are eased when the conductances are reduced.

**Figure 1:**
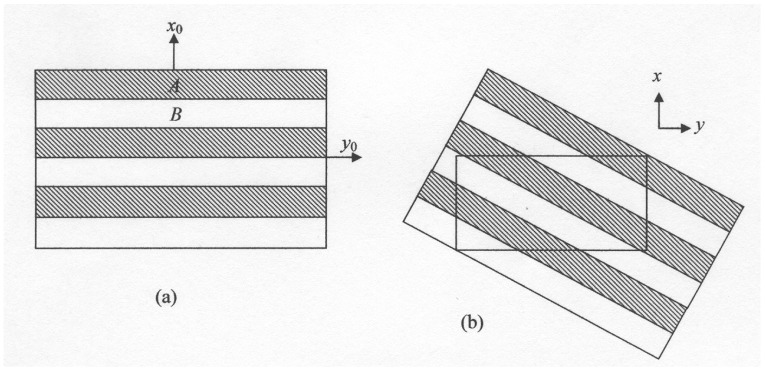
A synthetic anisotropic conductor. (a) Layered material consisting of 2 components. (b) Section cut to form a transverse thermoelement.

There has recently been renewed interest in the practical use of the transverse thermoelectric effects [4]. Transverse Seebeck and Peltier effects are observed in specimens that are cut at arbitrary orientations in substances that display anisotropic thermoelectric coefficients. These effects are small in all homogeneous samples but can become large in two-phase materials [5]. It has been demonstrated that effective transverse thermoelements can be cut from a layered material having the form shown in Figure 1. In the *x*_0_ direction the Seebeck coefficient is dominated by the component with the higher thermal resistance whereas in the *y*_0_ direction it is dominated by the component with the higher electrical conductance. We shall show that the performance of synthetic transverse thermoelements can be improved if one of the components is porous.

## 2. Porous Thermoelements in Conventional Modules

In theory the behaviour of a thermoelement should not change if the length and cross-section area are increased or decreased by the same proportion. However, as the dimensions of the thermoelements are reduced, the performance of the module in which they are incorporated is impaired. This is because of the increasing importance of electrical contact resistance and thermal gradients at the faces of the module. It is possible to reduce the thermal flux density by increasing the spacing between the thermoelements but there is then the possibility of heat losses by radiation and conduction in the unoccupied space [6]. An alternative is the use of porous thermoelements to reduce the amount of thermoelectric material in the module. This allows any radiation loss to be reduced since radiative heat flux is proportional to the difference between surface temperatures. It is clearly an advantage to insert numerous intermediate surfaces between the source and sink. It also alleviates the electrical contact resistance problem if such resistance resides in the joint material rather than the thermoelements.

The performance of a thermoelectric energy converter [7] can be expressed in terms of a figure of merit *Z* defined by the relation:

(1) $$Z=\frac{{\left(\alpha_{B}-\alpha_{A}\right)}^{2}}{{\left[{\left(\lambda_{B}\rho_{B}\right)}^{1/2}+{\left(\lambda_{A}\rho_{A}\right)}^{1/2}\right]}^{2}}$$

where *α* is the Seebeck coefficient, *λ* is the thermal conductivity, *ρ* is the electrical resistivity and the subscripts *A* and *B* denote the two branches.

The effect of electrical contact resistance is to increase the apparent electrical resistivity. Thermal resistance between the module and the source and sink, associated with a high flux density, decreases the coefficient of performance or the efficiency. Both these effects have been discussed previously with a view to minimising the volume of thermoelectric material by using widely spaced thermoelements of small length and cross-section [8]. Here we discuss the degradation of the figure of merit if porous material is employed.

**Figure 2:**
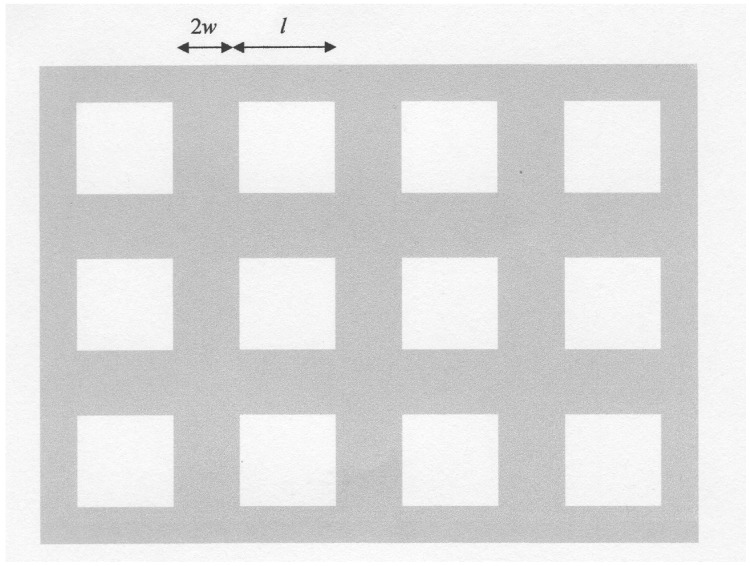
Model for the calculation of thermal losses in a porous material.

We make use of the model for a porous material shown in Figure 2. The pores are assumed to consist of cubic spaces of edge length *l* separated by material of width 2*w*. Each cell then consists of a cubic space surrounded by a wall of thickness *w*. It is assumed that the electrical and thermal flow is linear, an approximation that improves as the porosity becomes greater. It is appreciated that the pores in an actual material will probably be quite different from those in our model but the effect on the transport properties is likely to be realistic. One characteristic that will probably exist in any material with a high porosity is inter-connection between the pores. This feature does not exist in our model.

The heat losses will obviously be smallest if the pores are evacuated since then only radiative transfer will take place. The radiation loss can be reduced to any desired level by making the number of cells in a given volume as high as possible, so that the temperature difference between the opposite faces of a pore is very small. There will be applications in which evacuated pores can be realised but it has to be remembered that thermal conduction in a gas does not disappear until the mean free path of the molecules is much greater than the width of the containing vessel. This requires a very high vacuum. Inter-connection between the pores will assist in allowing the penetration of a vacuum or any filling gas throughout the material.

Usually the pores will be filled with a gas of thermal conductivity *λ_p_*, which will generally be much less than the thermal conductivity *λ* of the thermoelectric material. We then find that the thermal conductance of each cell is given by:

(2) $$K_{c}=\frac{\lambda l}{\lambda /\lambda_{p}+2w/l}+\frac{\lambda l\left[{\left(1+2w/l\right)}^{2}-1\right]}{1+2w/l}$$

If the thermal conductivity in the pores were equal to zero the thermal conductance per cell would be:

(3) $$K_{0}=\frac{\lambda l\left[{\left(1+2w/l\right)}^{2}-1\right]}{1+2w/l}$$

**Figure 3:**
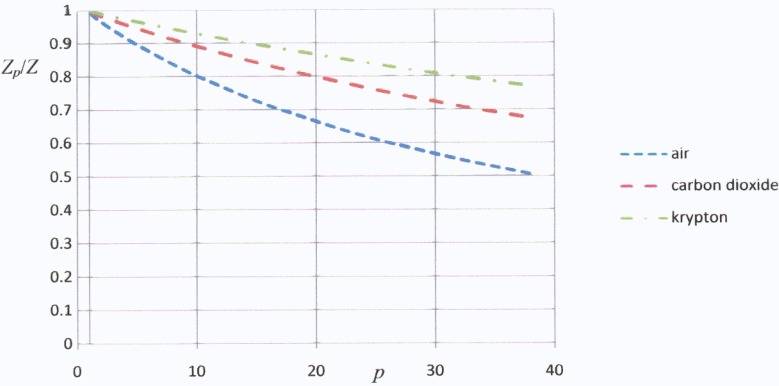
Plots of relative value for the thermoelectric figure of merit against the porosity factor for pores filled with air, carbon dioxide and krypton.

The ratio of the effective thermal conductivity of the porous material to that which would exist if the pores did not conduct the heat is *K_c_*/*K*_0_ and this is equal to the ratio of the figure of merit *Z_p_* of the porous material to that *Z* of a fully dense specimen. We have calculated this ratio for the pores being filled with air, carbon dioxide and krypton. The thermal conductivity of air is 0.026 W/m K. The losses are reduced by using carbon dioxide (*λ_p_* = 0.013 W/m K) instead of air and become still less if the filling gas is krypton (*λ_p_* = 0.008 W/m K) [9]. In Figure 3 we show how the figure of merit falls with the porosity factor *p* for these three cases. We define the porosity factor *p* as the ratio of the electrical conductivity in fully dense material to that of the porous material. Since the electrical conductivity is zero within the pores:

(4) $$p=\frac{{\left(l+2w\right)}^{2}}{{\left(l+2w\right)}^{2}-l^{2}}$$

The thermal conductivity of the fully dense thermoelectric material will, of course, depend on its composition. We shall assume that the material is a sintered bismuth telluride alloy for which a typical value of *λ* might be 1.0 W/m K. This value has been assumed in our calculations. 

Figure 3 shows that the figure of merit falls by about 20% for a porosity factor of 10 if the pores are filled with air but the decrease is less than 10% for the same porosity factor if the gas is krypton. Thus, provided that a suitable filling gas is used, it should be possible to reduce electrical contact resistance losses by an order of magnitude, with only a marginal fall in the effective figure of merit, by using porous thermoelectric material. It is noted that the porosity factor *p* is equal to 10 when the relative density is about 0.15.

## 3. Porous Components in Synthetic Transverse Thermoelements

We now turn to the inclusion of a porous component in a synthetic transverse thermoelement. It is supposed that the specimen is cut from a layered structure as shown in Figure 1. There will be a preferred ratio between the thicknesses of the layers and a preferred angle between the axes *x*,*y* and the axes *x*_0_*,y*_0_. One can then determine a transverse figure of merit *Z_φ_* that has the same significance in a transverse energy converter as does the figure of merit *Z* for a conventional thermocouple [4].

Let us assume that the electrical conductance of the *B* layers is much higher than that of the *A* layers in the *y*_0_ direction. Then the Seebeck coefficient in that direction will lie close to its value for the *B* layers. We also suppose that the thermal resistance in the *x*_0_ direction is dominated by the *A* layers. Then the Seebeck coefficient in that direction will be nearly equal to that of component *A*. It is most desirable that the Seebeck coefficient should be very different and preferably of opposite sign in the two components. It is also necessary that the two materials, *A* and *B,* should have a high thermoelectric figure of merit *Z* when used as a conventional thermocouple [10]. The transverse figure of merit is always less than *Z,* because of internal thermoelectric eddy currents, but the difference may be quite small.

In the past, it has been difficult to satisfy these requirements using fully dense materials. The best conventional couples for use at ordinary temperatures make use of p-type and n-type bismuth telluride alloys [8]. However, these materials have very similar values for the electrical and thermal conductivity. This prevents us from simultaneously making the electrical conductance of, say, component *B* much larger than that of component *A* in one direction and the thermal resistance of component *A* much the larger in the other direction. One really requires *σ_A_λ_B_*/*σ_B_λ_A_* >> 1. Kyarad and Lengfellner [5] resolved this dilemma by using a thermoelectric semiconductor, bismuth telluride, as one component and a metal, lead, as the other. However, in doing so they sacrificed the thermocouple figure of merit. Even so, they were able to obtain 22º of cooling using the transverse Peltier effect. Marginally better results were achieved earlier by Gudkin et al. [11] using a bismuth telluride alloy in conjunction with bismuth. Gudkin and his colleagues also demonstrated one of the major advantages of transverse thermoelectric cooler, i.e. the capacity for making an infinite cascade by suitable shaping of the thermoelement. Cooling through 23º using a rectangular block was increased to 35º using a trapezoidal cross-section.

We have shown previously [12] that the condition *σ_A_λ_B_*/*σ_B_λ_A_* >> 1 can be achieved in principle when both components consist of bismuth telluride alloys if one of them is porous. However, it would be necessary to have an extremely high porosity with no significant heat transfer through the pores. A high transverse figure of merit could be reached much more easily by combining porous p-type sintered bismuth-antimony telluride with the metallic conductor, YbAl_2.96_Mn_0.04_, which has the reasonably high negative Seebeck coefficient of -90 μV/K [13]. We have found that this combination should yield a transverse dimensionless figure of merit *Z_φ_T* equal to almost 0.7 when the porosity factor *p* in the p-type component is about 10 and when the pores are evacuated. 

**Figure 4:**
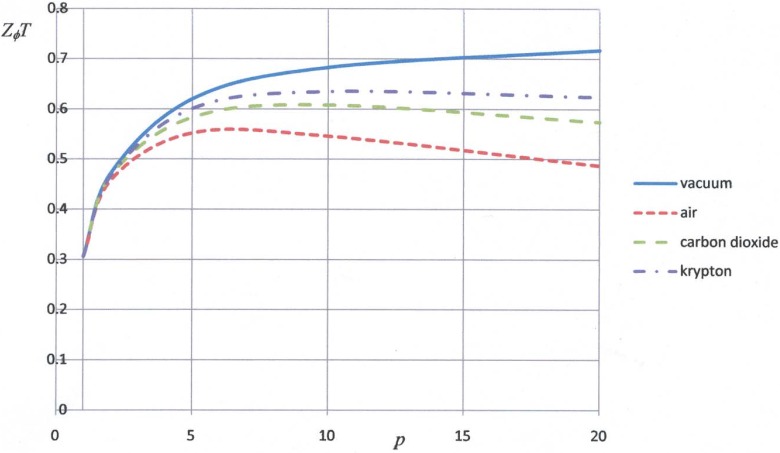
Plots of the transverse figure of merit against the porosity factor for pores filled with air, carbon dioxide and krypton. The components of the transverse thermoelement are porous sintered (Bi,Sb)_3_ and dense YbAl_2.96_Mn_0.04_.

We have now determined the variation of the transverse figure of merit with the porosity of the p-type component taking into account heat conduction within the pores. The results are shown in Figure 4.

It is apparent that porosity in the p-type component produces a substantial increase in the transverse figure of merit even when the pores are filled with air. In this case, the maximum value of *Z_φ_T* is equal to about 0.56 when the porosity factor is 6. Values of *Z_φ_T* in excess of 0.6 should be achieved for a porosity factor of about 10 if the pores are filled with carbon dioxide or krypton. This means that a rectilinear thermomagnetic cooler made from porous bismuth-antimony telluride and YbAl_2.96_Mn_0.04_ operating from a heat sink at 300 K should be able to achieve a maximum temperature depression in excess of 60º. In view of the ease with which an infinite-staged cascade can be produced using a shaped transverse thermoelement, it is likely that a temperature depression of more than 100º might be achieved using a positive component with evacuated pores.

## 4. Conclusions 

We have shown that there may be a practical advantage of economical use of material without reduction in performance if porous thermoelectric materials are used in the fabrication of modules. However, the most important use of such materials is likely to be in synthetic transverse thermoelements. The transverse thermoelectric figure of merit reaches a substantially higher value if a fully dense bismuth-antimony telluride alloy is replaced by material with a porosity factor of about 10. The improvement is greatest if the pores are evacuated but is still large if the pores are gas-filled particularly if a gas with a low thermal conductivity, such as krypton, is employed.
